# Detection of *Trypanosoma cruzi* DNA in Blood of the Lizard *Microlophus atacamensis*: Understanding the *T. cruzi* Cycle in a Coastal Island of the Atacama Desert

**DOI:** 10.3390/ani15091221

**Published:** 2025-04-26

**Authors:** Josefa Borcosque-Avendaño, Nicol Quiroga, Franco Cianferoni, Gabriel Díaz-Campusano, José Luis Marcos, Carezza Botto-Mahan, Fernando Torres-Pérez, Antonella Bacigalupo, Ricardo Campos-Soto

**Affiliations:** 1 Facultad de Ciencias de la Vida, Universidad Viña del Mar, Viña del Mar 2572007, Chile; jborcosqa@gmail.com (J.B.-A.); jmarcos@uvm.cl (J.L.M.); 2 Departamento de Ciencias Ecológicas, Facultad de Ciencias, Universidad de Chile, Santiago 7800003, Chile; nicol.quiroga.h@gmail.com (N.Q.); cbotto@uchile.cl (C.B.-M.); 3 Instituto de Biología, Facultad de Ciencias, Pontificia Universidad Católica de Valparaíso, Valparaíso 2373223, Chile; franco.cianferoni.a@gmail.com (F.C.); gabrieldiazcampusano@gmail.com (G.D.-C.); fernando.torres@pucv.cl (F.T.-P.); 4 Research Ring in Pest Insects and Climatic Change (PIC2), Universidad de Chile, Santiago 7800003, Chile; 5 School of Biodiversity, One Health and Veterinary Medicine, University of Glasgow, Glasgow G12 8QQ, Scotland, UK; anto.e.bacigalupo@gmail.com

**Keywords:** *Trypanosoma cruzi*, reptiles, wild *T. cruzi* cycle, *Microlophus*, Chagas disease

## Abstract

*Trypanosoma cruzi*, the protozoan causative of Chagas disease, is primarily transmitted through blood-sucking insects and infects mammalian and some reptilian hosts. In Chile, insects of the *Mepraia* genus are key vectors of *T. cruzi* in its wild transmission cycle. High prevalence and mixed infection of *T. cruzi* lineages have been reported in a *Mepraia* population on Santa María Island in the Atacama Desert. However, no small mammals have been reported. The island’s vertebrate community is dominated by the lizard *Microlophus atacamensis* and marine and scavenger birds. This study aimed to research blood samples of *M. atacamensis* for the presence of *T. cruzi* DNA (kDNA and satDNA) using conventional PCR (cPCR) and quantitative real-time PCR (qPCR) and estimate parasitemia. Our findings reveal that 39.4% of 33 individuals were positive with both cPCR and qPCR, while when assessing infection with either technique, it rises up to 81.8%. These findings confirm that *M. atacamensis* is a host of *T. cruzi*, suggesting its potential role as a key reservoir in the island’s transmission cycle. This study provides new insights into the life cycle of *T. cruzi* in the coastal Atacama Desert, highlighting the importance of reptiles in the epidemiology of this parasite.

## Simple Summary

Chagas disease is caused by the protozoan parasite *Trypanosoma cruzi*. The cycle of this parasite includes blood-sucking insects as vectors (triatomines), as well as mammals and reptiles. *Mepraia* spp. are triatomines that transmit *T. cruzi* in the wild cycle of Chile. This study aims to detect *T. cruzi* in the blood of the lizard *Microlophus atacamensis*, endemic to a coastal island of the Atacama Desert characterized by a high prevalence of *T. cruzi* in triatomines and low abundance of mammals. Our findings reveal that *M. atacamensis* lizards are infected with *T. cruzi*. These results are essential to understanding the life cycle of *T. cruzi* in this particular island of the Atacama Desert and provide new knowledge to the epidemiology of Chagas disease.

## 1. Introduction

The One Health concept includes wild and domestic animals, the ecosystem, biodiversity and humans as interrelated parts to achieve a global wellness [1,2,3], and is the current way to understand and manage zoonotic diseases at a global scale. Chagas disease is one of the most important zoonoses mediated by vectors in Latin America. It is estimated that 6–7 million people are infected and 75 million are at risk of infection [4,5]. This disease is caused by the protozoan parasite *Trypanosoma cruzi*; the cycle of this parasite includes blood-sucking insects of the subfamily Triatominae [6], which serve as vectors and infect mammalian and reptile hosts [6,7,8,9]. Currently, the *T. cruzi* taxon is divided into seven discrete typing units (DTUs) named TcI to TcVI and Tcbat [10,11,12].

In Chile, triatomines of the genus *Mepraia* are part of the wild transmission cycle of *T. cruzi* as vectors. The genus *Mepraia* is endemic to semiarid and arid regions, and is distributed across coastal and interior valleys of northern and central Chile [13,14]. *Mepraia* currently includes three species: *M. spinolai*, *M. gajardoi* and *M. parapatrica*. The latter two are restricted to the coastal Atacama Desert between 18° and 26° S, while the former is mainly distributed in the interior valleys from 26° to 34° S [13,14,15]. Recent studies proposed that triatomines inhabiting the coast of the Antofagasta Region (Mejillones peninsula), located between the distributions of *M. parapatrica* and *M. gajardoi*, represent a new lineage, *Mepraia* sp., whose taxonomic status remains unsolved [14].

Island populations of *M*. *parapatrica* and *Mepraia* sp. have been reported infected with *T. cruzi* on Pan de Azúcar Island (Atacama Region) and Santa María Island (Antofagasta Region), respectively [16,17]. On Pan de Azúcar Island, molecular analyses using quantitative real-time PCR (qPCR) and cytochrome b sequencing revealed that triatomines feed on reptiles (*Microlophus atacamensis*), scavenger birds (*Cathartes aura*) and mammals (*Homo sapiens*, *Abrothrix olivaceus* and *Mus musculus*) [18]. Furthermore, on Pan de Azúcar Island, *T. cruzi* was detected by conventional PCR (cPCR) and qPCR in *A. olivaceus*, with an infection frequency of 6.2%, confirming the presence of a transmission cycle involving triatomines and mammalian hosts [17]. On the other hand, on Santa María Island, even though no rodents have been captured, high *T. cruzi* infection frequency (28.9% by cPCR, 36.8% by qPCR) was reported in triatomines [17].

Mixed infections, involving more than one DTU of *T. cruzi* within the same triatomine, were also reported on Santa María Island [16]. Such mixed infections are more commonly reported in interior valleys of central Chile, where a higher diversity of mammalian hosts may facilitate the circulation of multiple *T. cruzi* DTUs [19,20].

The vertebrate community on Santa María Island is dominated by reptiles, particularly *M. atacamensis*, alongside marine and scavenger birds. These observations suggest that *M. atacamensis* may serve as a host and play a role in the *T. cruzi* transmission cycle on the island. Although nuclear satellite DNA (satDNA) of *T. cruzi* has been detected in some reptile species, including *M. atacamensis* [8], the parasite has not yet been confirmed in lizards using kinetoplast DNA (kDNA) as target molecule, and parasitemia has not been estimated either. This study aims to examine the presence and quantify *T. cruzi* in the blood of *M. atacamensis* from Santa María Island using cPCR and qPCR to amplify kDNA and satDNA segments, respectively.

## 2. Materials and Methods

### 2.1. Study Area and Sample Collections in Lizards

**Figure 1:**
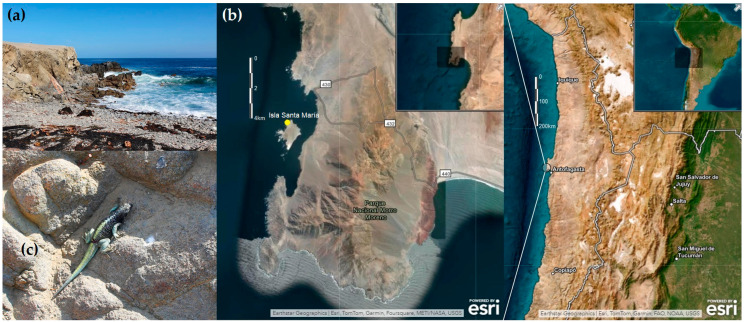
(**a**) Landscape of capture site on Santa María Island. (**b**) Map showing the capture site of *Microlophus atacamensis* with a yellow circle within Santa María Island in Mejillones peninsula, Chile, South America. (**c**) Individual of *M. atacamensis* from Santa María Island. Photographs (**a**,**c**) were taken by Ricardo Campos-Soto. The map was constructed using the ArcGIS Online platform [23].

*Microlophus atacamensis* lizards were collected during the austral summer of 2021 on Santa María island (23°25′53″ S, 70°36′33″ W) located off the coast of the Mejillones peninsula in the Antofagasta Region (Figure 1). This locality is included within the Atacama Desert and is characterized by an arid climate with oceanographic influence [21]. Lizards were manually captured using a rope with a slipknot. A total of 34 *M. atacamensis* were captured during the sampling period and blood samples were successfully collected from 33 individuals; one juvenile lizard (ID M11) was excluded from sampling due to its developmental stage. Blood samples (100 to 150 μL) were collected from the ventral coccygeal vein utilizing an insulin syringe with a 29G needle after dose–effect sedation with isoflurane [22]. The blood samples were immediately transferred to cryotubes and stored in a liquid nitrogen container. All the lizards were marked with a temporal nontoxic highlighter for traceability, and once recovered from isoflurane, they were released at their respective capture points (Figure 1c).

### 2.2. DNA Extraction from Blood Samples of Lizards

DNA was extracted from 100 µL blood samples using the Blood and Tissue Kit DNeasy^®^ (QIAGEN, Hilden, Germany) following the manufacturer’s instructions. To detect DNA loss or PCR inhibition, each sample was co-extracted with 100 pg of a sequence of 183 base pairs (bp) of the tonoplast intrinsic protein from *Arabidopsis thaliana*, used as a heterologous internal amplification control (IAC) [24]. Finally, the DNA was eluted twice with 100 µL of elution buffer and stored at −20 °C.

### 2.3. Kinetoplast DNA Amplification of T. cruzi by Conventional PCR

A segment of 330 base pairs (bp) of the variable region of the minicircle kinetoplast DNA (kDNA) of *T. cruzi* was amplified using primers 121 and 122 [25,26]. The amplification reaction was conducted in a thermal cycler Bioer model TC-96/G/H(b)C LifeEco^®^ (Hangzhou, China), using the polymerase SapphireAmp^®^ fast PCR Master Mix (Takara, Kusatsu, Japan) and cycling parameters as reported [26]. Each reaction included a positive control which consisted of DNA extracted from *T. cruzi* culture (strain DM28c TcI) and a no-template control (nuclease-free water). Before cPCR, a diluted aliquot was prepared using 2 μL of the extracted DNA and 18 μL of nuclease-free water to prevent PCR inhibition due to excess DNA. Due to the large amount of lizard DNA, each sample was tested in duplicate using 2 µL diluted aliquot DNA. The amplification success was confirmed by visualizing a 330 bp band on a 3% agarose gel, and was considered infected with *T. cruzi* when at least one duplicate amplified the kDNA. Negative samples were retested under the same conditions and classified as positive if at least one replicate yielded a positive result.

### 2.4. Satellite DNA Amplification of T. cruzi by Quantitative PCR

A segment of the nuclear satDNA of *T. cruzi* was amplified using the primers Cruzi 1, Cruzi 2 and the probe Cruzi 3 [27], 1× Luna Universal Probe qPCR Master Mix (New England Biolabs, Ipswich, MA, USA), 0.45 μM of each primer (Cruzi 1 and 2), probe at 0.15 μM and 2 μL of diluted DNA reaching a final volume of 20 μL. All the assays were performed in a QuantStudio 3 Real-Time PCR System (Applied Biosystems, Foster, CA, USA). The thermal profile consisted of an initial denaturation phase of 60 s at 95 °C, followed by 40 denaturation cycles of 15 s at 95 °C, 30 s at 60 °C [28]. Due to the large amount of lizard DNA, samples were tested in diluted form (2 μL of sample and 18 μL of nuclease-free water). Each assay included a positive control with DNA from *T. cruzi* cultures TCII-CDMC and nuclease-free water as a no-template control. A real-time PCR was also performed for each sample to detect the IAC following the reported conditions [18,29]. Each sample was tested in duplicate and was considered positive if: (i) it amplified both duplicates with a cycle threshold (Ct) ≤ 38, and (ii) the IAC was efficiently amplified. Samples with one positive replicate and the second negative, i.e., with no Ct, or with a Ct > 38, were considered inconclusive (ic).

To perform absolute quantification, a standard curve was generated by mixing DNA from cultured *T. cruzi* (TCII-CDMC) and DNA from *T. cruzi*-free chicken liver, the latter being the closest tissue available to a lizard. The *T. cruzi* DNA was diluted six times in chicken DNA to obtain a curve from 5 × 10^6^ to 5 × 10^1^ par-eq/mL, and the last concentration was taken as the limit of quantification [30,31]. The standard curve parameters were: Ct = 0.055, efficiency = 96.5%, and linear regression coefficient, R^2^ = 0.995 (Figure S1). The qPCR analysis was performed in Quantstudio™ Design & Analysis Software v1.5.2.

### 2.5. Data Analysis

The results from both amplification techniques (kDNA and satDNA) were subjected to Cohen’s Kappa test to measure their agreement using the web platform VassarStats http://vassarstats.net/index.html (accessed on 28 March 2025). For this comparison, inconclusive qPCR results were assumed to be negative.

### 2.6. Sequencing of the Amplified satDNA Segments

We randomly selected seven samples positive to both cPCR and qPCR, and the replicate with Ct ≤ 38 from an inconclusive qPCR sample with negative cPCR, for sequencing. The products of those qPCRs (amplified segments of satDNA) were submitted to Macrogen https://www.macrogen.com (accessed on 12 March 2025) for both forward and reverse strand sequencing, employing the same qPCR primers Cruzi 1 and Cruzi 2 [27]. The sequences were edited with visual inspection of the chromatogram using BioEdit 7.2.5 [32], and the obtained consensus sequences were deposited in GenBank^®^ with accession numbers PV297859–PV297866. Each sequence was compared with those available in GenBank^®^ using the alignment algorithm BLASTN 2.16.1+ implemented in the online BLAST^®^ platform [33,34].

## 3. Results

### 3.1. kDNA and satDNA Amplification

**Figure 2:**
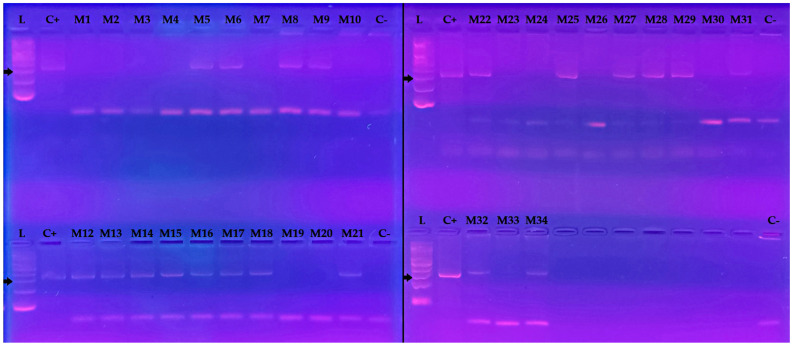
Agarose gel electrophoresis of conventional PCR product of kDNA of *T. cruzi* in blood samples of *Microlophus atacamensis* from Santa María Island. Lanes L: 100-bp DNA ladder; C+: Positive control; M1 to M34 are the ID samples; C−: non-template control. Black arrows indicate 300 bp. Photo credit: Josefa Borcosque. Original images of the electrophoresis gels are shown in Figure S2.

Using conventional PCR targeting the kinetoplast kDNA of *T. cruzi*, we detected the presence of the parasite’s kDNA in 20 out of 33 lizards, corresponding to an infection prevalence of 60.6% (Figure 2).

Quantitative PCR revealed infection in 20 lizards, representing a prevalence of 60.6%. All samples amplified the IAC. Raw data of Ct values for *T. cruzi* detection and IAC amplification are shown in Supplementary Tables S1 and S2, respectively. Parasite quantification was only possible for sample M22, with an estimated mean parasite load ± sd of 572.0 ± 55.0 par-eq/mL.

Table 1 summarizes the infection status among the sampled individuals, revealing that 13 of the sampled lizards (39.4%) were positive to *T. cruzi* by both cPCR and qPCR methods. The value for Cohen’s Kappa between cPCR and qPCR was 0.11, and according to the Landis and Koch table [35], this value indicates slight agreement. Considering that at least one method is enough to consider an individual positive, the infection frequency in the sampled *M. atacamensis* was 27/33 (81.8%).

### 3.2. Sequencing of the satDNA Segments

All satDNA sequences matched with *T. cruzi* in the BLAST^®^ analysis as the first match (Table 2). The principal BLAST indices (Score, percent identity, E value, and Query Cover) show significant alignments. The higher match for each sample and their indices are shown in Table 2.

## 4. Discussion

This study aimed to detect *T. cruzi* in lizards’ blood from Santa María Island using cPCR and qPCR to amplify kDNA and satDNA segments, respectively. We found that many of the analyzed *M. atacamensis* individuals were positive to *T. cruzi*, but the results were not always concordant between techniques.

Reptiles can host several trypanosomatid species, which are mainly transmitted by sandfly dipterans [36,37,38]. Some of the trypanosomatids reported in reptiles may have zoonotic importance, such as *Leishmania*, *T. brucei* and *T. cruzi,* which was recently reported [8,9,36,39].

In Chile, on Pan de Azúcar Island the complete life cycle of *T. cruzi* involving small mammals and triatomines was described [17]. However, on Santa María Island, the absence of captured rodents, coupled with high infection frequencies in triatomines (28.9% by cPCR, 36.8% by qPCR), suggested a different transmission dynamic [17]. It has been reported that Chilean lizards [8] and Brazilian snakes can be hosts of *T. cruzi* [9]. Given that *M. atacamensis* is abundant within the vertebrate community on Santa María Island, its role in maintaining the *T. cruzi* cycle may be crucial.

Advances in genomics and PCR techniques make it easier to detect parasite DNA, which is now used as a marker for infection [8,9,40,41]. Our findings provide strong evidence that *M. atacamensis* lizards on Santa María Island are infected with *T. cruzi*. We detected both kDNA and satDNA of *T cruzi* in 39.4% of the sampled lizards (Table 1). Multi-target detection of *T. cruzi* is encouraged for robust results [42,43]. The combined use of these markers is considered a reliable approach for *T. cruzi* detection, as it minimizes the risk of cross-reactivity with other trypanosomes, such as *T. rangeli* [43]. For example, cross-reactivity in kDNA and nuclear DNA detection has been reported between *T. cruzi* and *T. rangeli* [43,44]. *Trypanosoma rangeli* is transmitted by triatomines of the genus *Rhodnius* and is also present in *Panstrongylus* [45]. These triatomine genera are not present in the Atacama Desert of Chile; therefore, it is very unlikely that these results represent a cross-reactivity with *T. rangeli*. Also, the BLAST analysis result shows that the satDNA sequences correspond to *T. cruzi* and not to other *Trypanosoma* species.

The high detection rate of kDNA by cPCR (Table 1) is consistent with previous studies, and is unsurprising given the amplification protocol used [26] and its high sensitivity [43,44]. This sensitivity is attributed to the high copy numbers of kDNA, with estimates ranging from 5000 to 30,000 minicircles per parasite and between 20,000 and 120,000 copies of the variable region of the minicircle [43,46,47]. On the other hand, it cannot be ruled out that the amplified minicircle corresponds to a reptilian *Trypanosoma* or to an undescribed trypanosomatid. There are several described trypanosomes of lizards and snakes [9,37,38,48], most of which belong to the established subgenus *Squamatrypanum* [49]. However, in this study, the significant results of the BLAST analysis of the satDNA sequences support *T. cruzi* as the infecting trypanosomatid (Table 2).

In some samples subjected to both tests, only kDNA or satDNA was amplified (Table 1). The calculated Cohen’s Kappa coefficient of 0.11 indicates slight agreement between both tests. This discrepancy may be related to the probability of obtaining amplifiable kDNA or satDNA from the samples and potential variations in sample quality. In fact, the copy number of satDNA varies among different *T. cruzi* DTUs [46,50]. Furthermore, the DTU TcI shows significant genetic diversity [51,52]. Consequently, certain variants may possess fewer satDNA copies or reduced primer complementarity, further contributing to the observed differences in amplification. Also, as it was recently mentioned, it cannot be ruled out that this discrepancy can be due to an undescribed variant of *T*. *cruzi* or to an undescribed trypanosomatid.

We could quantify *T. cruzi* only in one sample (M22); to the best of our knowledge, this is the first parasite load estimation in a lizard. Parasite loads are difficult to estimate in the lizards’ blood due to the presence of nucleated erythrocytes, which tend to inhibit the PCR due to excess DNA [9]. We overcame PCR inhibition by sample dilution, but in the process, several samples did not reach the quantifiable limit of our curve.

Although this study reports the presence of *T. cruzi* DNA in the blood of *Microlophus* lizards, the mechanism of infection remains unverified. Oral transmission is a plausible route, as these lizards prey on insects [53], including triatomines [17].

Identifying a new natural reservoir requires an evaluation of its host competence, defined as the ability of a host species to transmit the parasite to another susceptible host species or vector and maintain the infection [54,55,56]. While the host competence of *Microlophus* in transmitting *T. cruzi* to triatomines in coastal areas is currently unknown, previous studies using xenodiagnoses have demonstrated host competence in wild lizard *Liolaemus platei* from arid–semiarid Mediterranean regions of Chile [8]. Regarding birds, although *T. cruzi* DNA has been detected in bird tissues [41], currently, there is no evidence that they are competent hosts.

*Microlophus atacamensis* inhabits the intertidal zones of the Atacama Desert from 22°10′ S to 29°41′ S [57,58], so future studies should consider evaluating its infection with *T. cruzi* along its whole distribution area.

Similar ecological characteristics to those present in Santa María Island, where reptiles and triatomines coexist, are present in several coastal zones of Chile [17,18,19,59]. In these coastal areas, an increasing population of seaweed collectors, fishermen and immigrants live in vulnerable conditions due to unawareness of the danger that triatomines represent [18,59]. People bring their pets; dogs and cats may hunt infected lizards and become orally infected [5,60,61,62], posing a potential transmission risk to triatomines near households. Therefore, it is necessary to evaluate the infection prevalence of *T. cruzi* in lizards from different regions of northern Chile, and to assess their host competence to determine the associated transmission risk to domestic animals. Consumption of reptiles by people of these areas is not reported, and reptiles are protected species in Chile, but in case of occurrence, it would involve a *T. cruzi* transmission risk as well.

By providing evidence of *T. cruzi* infection in *M. atacamensis*, this research contributes to a deeper understanding of the parasite’s life cycle in the coastal Atacama Desert, highlighting reptiles’ potential role in its eco-epidemiology.

## 5. Conclusions

This study reports high *T. cruzi* prevalence, detecting kDNA, satDNA and quantifying this parasite in the blood of *M*. *atacamensis*, proving that this lizard species is a host of *T. cruzi*, and could be an important *T. cruzi* reservoir in coastal areas of the Atacama Desert. This finding contributes to a better understanding of the life cycle of *T. cruzi* in these areas and provides new knowledge about the eco-epidemiology of Chagas disease.

## Figures and Tables

**Table 1:** *Trypanosoma cruzi* detection in blood of *Microlophus atacamensis* by amplification of kDNA using conventional PCR and by amplification of nuclear satDNA using qPCR.

Lizard ID	cPCR	qPCR
M1	−	ic
M2	−	+
M3	−	+
M4	−	+
M5	+	+
M6	+	−
M7	−	ic
M8	+	+
M9	+	+
M10	−	ic
M12	+	+
M13	+	+
M14	+	+
M15	+	+
M16	+	ic
M17	+	+
M18	+	+
M19	−	ic
M20	−	+
M21	+	+
M22	+	+
M23	−	+
M24	−	ic
M25	+	ic
M26	−	+
M27	+	+
M28	+	ic
M29	+	−
M30	−	ic
M31	+	ic
M32	+	+
M33	−	+
M34	+	ic
Total	20/33 (60.6%)	20/33 (60.6%)

cPCR: Conventional PCR, qPCR: Quantitative Real-Time PCR, +: positive sample, −: negative sample, ic: inconclusive. The infection percentage by each technique is shown in parentheses. Lizard M11 was excluded from sampling due to its juvenile developmental stage.

**Table 2:** BLAST analysis results of SatDNA sequences amplified from blood samples of the lizard *Microlophus atacamensis*.

Lizard ID	Sequence Length (bp)	Accession Number	Score	Query Cover (%)	E Value	Identity (%)	Species Match
M5	82	PV297859	135	100	5 × 10^−28^	96.34	*Trypanosoma cruzi*
M13	96	PV297860	158	95	1 × 10^−34^	97.80	*Trypanosoma cruzi*
M14	164	PV297861	270	98	3 × 10^−68^	96.89	*Trypanosoma cruzi*
M15	110	PV297862	145	98	1 × 10^−30^	90.74	*Trypanosoma cruzi*
M18	164	PV297863	255	99	1 × 10^−63^	95.06	*Trypanosoma cruzi*
M19	168	PV297864	268	95	1 × 10^−67^	96.88	*Trypanosoma cruzi*
M20	108	PV297865	167	100	3 × 10^−37^	94.44	*Trypanosoma cruzi*
M27	108	PV297866	150	100	3 × 10^−32^	91.67	*Trypanosoma cruzi*

## Data Availability

The data presented in this study are available in the manuscript and in its Supplementary Material (Tables S1 and S2, Figures S1 and S2).

## Supplementary Materials

The following supporting information can be downloaded at: https://www.mdpi.com/article/10.3390/ani15091221/s1, Table S1: Cycle threshold (Ct) values of quantitative real-time PCR (qPCR) assay for *Trypanosoma cruzi* amplification in blood samples of *Microlophus atacamensis*; Table S2: Cycle threshold (Ct) values of real-time PCR essay for the internal amplification control (IAC); Figure S1: Graph of the parasite standard amplification curve; Figure S2: Original images of the electrophoresis gels.

## Abbreviations

The following abbreviations are used in this manuscript:

cPCR	Conventional PCR
qPCR	Quantitative Real-Time PCR
satDNA	Satellite DNA
kDNA	Kinetoplast DNA
IAC	Internal amplification control
Ct	Cycle threshold
