# Supplementary material for: Detection of Trypanosoma cruzi DNA in Blood of the Lizard Microlophus atacamensis: Understanding the T. cruzi Cycle in a Coastal Island of the Atacama Desert
Source: Animals (Basel). 2025 Apr 26;15(9):1221. doi: 10.3390/ani15091221 (PMC12071183; doi:10.3390/ani15091221)
Supplement: Supplementary file 1 [file animals-15-01221-s001.zip › animals-3490826-supplementary.pdf]

Table S1. Cycle threshold (Ct) values of quantitative real-time PCR (qPCR) assay for *Trypanosoma cruzi* amplification in blood samples of *Microlophus atacamensis*.

Sample ID	Ct a	Ct b	Ct Mean	Ct SD
Mi1-dil10	38.7	36.5	37.6	1.6
Mi2-dil10	35.7	35.2	35.4	0.4
Mi3-dil10	35.0	37.2	36.1	1.5
Mi4-dil10	33.7	33.4	33.5	0.2
Mi5-dil10	33.9	34.9	34.4	0.8
Mi6-dil10	No Ct	No Ct		
Mi7-dil10	37.1	No Ct	37.1	
Mi8-dil10	35.9	36.2	36.1	0.3
Mi9-dil10	36.8	35.9	36.3	0.7
Mi10-dil10	37.6	38.3	38.0	0.4
Mi12-dil10	35.5	37.1	36.3	1.1
Mi13-dil10	34.1	34.2	34.1	0.1
Mi14-dil10	35.7	34.3	35.0	1.0
Mi15-dil10	34.9	34.8	34.8	0.1
Mi16-dil10	No Ct	34.7	34.7	
Mi17-dil10	36.9	37.4	37.2	0.3
Mi18-dil10	37.7	37.3	37.5	0.3
Mi19-dil10	No Ct	36.1	36.1	
Mi20-dil10	36.4	36.9	36.7	0.4
Mi23-dil10	34.5	349	34.7	0.3
Mi24-dil10	No Ct	36.5	36.5	
Mi25-dil10	36.6	No Ct	36.6	
Mi26-dil10	35.4	34.9	35.2	0.3
Mi27-dil10	35.0	34.2	34.6	0.5
Mi28-dil10	36.3	38.3	37.3	1.5
Mi30-dil10	38.9	37.6	38.3	0.9
Mi31-dil10	No Ct	35.4	35.4	
Mi32-dil10	34.3	34.1	34.2	0.2
Mi33-dil10	36.0	37.4	36.7	1.0
Mi34-dil10	38.0	36.4	37.2	1.2
Positive control	22.9	23,0	22.9	0.0
NTC	No Ct	No Ct		
MI 22-dil10	32.9	32.7	32.8	0.1
MI 21-dil10	33.5	33.5	33.5	0.0
MI 29-dil10	No Ct	No Ct		
Positive control	22.7	23.0	22.9	0.2
NTC	No Ct	No Ct		

SD: standard deviation, No Ct: without amplification

Table S2. Cycle threshold (Ct) values of real-time PCR essay for the internal amplification control (IAC). SD: standard deviation.

Sample ID	Ct a	Ct b	Ct Mean	Ct SD
Mi 1	19.7	19.5	19.6	0.1
Mi 2	20.8	20.7	20.7	0.1
Mi 3	19.8	19.7	19.8	0.0
Mi 4	20.9	21.1	21.0	0.1
Mi 5	20.0	19.6	19.8	0.3
Mi 6	20.3	20.2	20.2	0.1
Mi 7	21.0	20.8	20.9	0.1
Mi 8	20.0	19.9	19.9	0.1
Mi 9	20.3	19.5	19.9	0.6
Mi 10	20.2	19.4	19.8	0.6
Mi 12	18.8	18.6	18.7	0.1
Mi 13	19.2	19.4	19.3	0.1
Mi 14	36.0	19.7	27.8	11.6
Mi 15	19.2	19.0	19.1	0.1
Mi 16	18.8	19.0	18.9	0.1
Mi 17	19.4	19.4	19.4	0.0
Mi 18	17.8	17.9	17.8	0.1
Mi 19	23.2	23.4	23.3	0.2
Mi 20	19.7	19.6	19.7	0.1
Mi 21	18.3	18.4	18.4	0.1
Mi 22	24.0	24.0	24.0	0.0
Mi 23	23.4	23.5	23.4	0.1
Mi 24	22.5	22.9	22.7	0.3
Mi 25	23.0	23.1	23.1	0.1
Mi 26	22.4	22.6	22.5	0.1
Mi 27	19.3	19.3	19.3	0.0
Mi 28	23.7	23.7	23.7	0.0
Mi 29	23.8	23.9	23.9	0.1
Mi 30	23.0	23.1	23.0	0.0
Mi 31	23.7	23.8	23.7	0.1
Mi 32	23.6	23.5	23.5	0.1
Mi 33	24.0	24.0	24.0	0.0
Mi 34	24.0	24.1	24.1	0.0

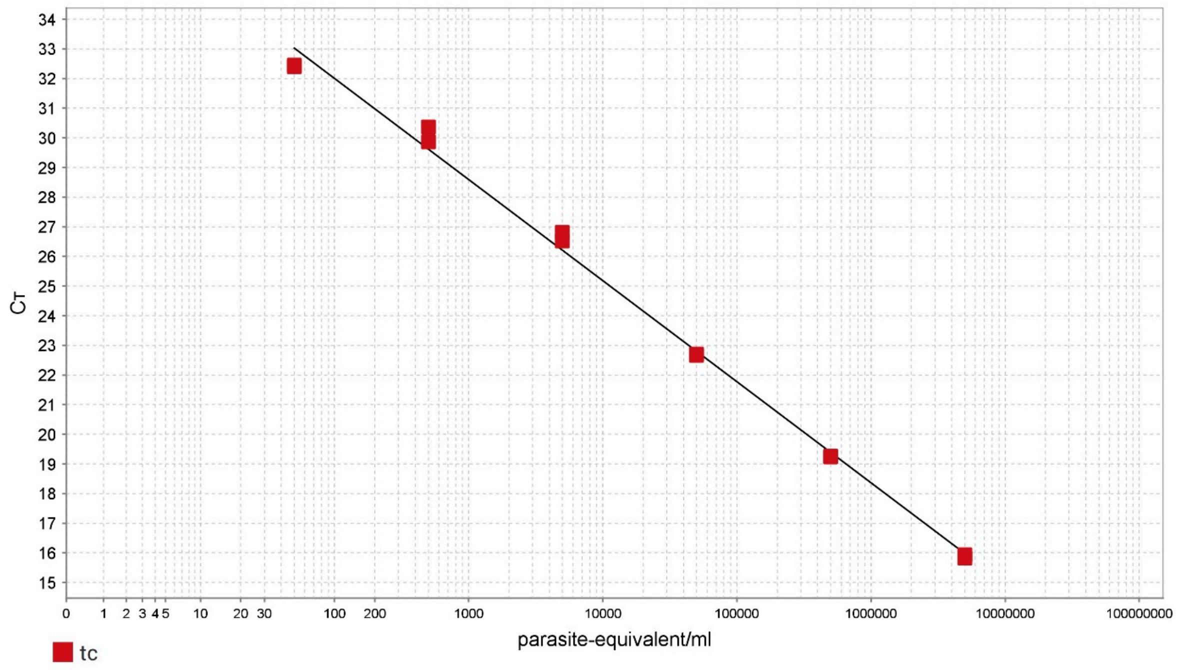

Figure S1. Graph of the parasite standard amplification curve. Linear regression of the standard amplification curve generated by 6 serial fold dilutions of DNA from chicken liver and with *T. cruzi* DNA ($5x10^6$ to $5x10^1$ par-eq/ml). The parameters of the curve are Ct = 0.055. efficiency % = 96.5 and linear regression coefficient. R^2 = 0.995.

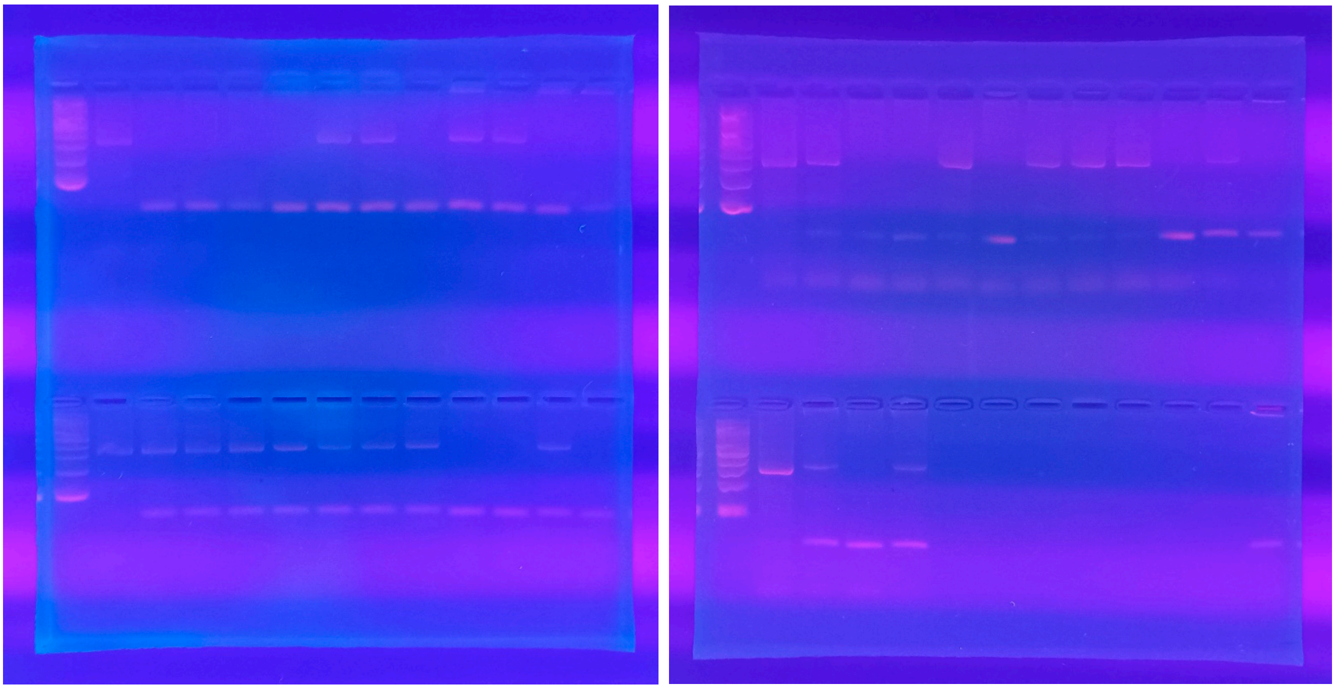

Figure S2. Original images of the electrophoresis gels. The left photograph shows the electrophoresis of the PCR products of samples 1-21 while the right photograph shows samples 22-34. according to Figure 2. Photo credit: Josefa Borcosque
